# High Throughput Screening of Additives Using Factorial Design to Promote Survival of Stored Cultured Epithelial Sheets

**DOI:** 10.1155/2018/6545876

**Published:** 2018-11-18

**Authors:** Sjur Reppe, Catherine Joan Jackson, Håkon Ringstad, Kim Alexander Tønseth, Hege Bakke, Jon Roger Eidet, Tor Paaske Utheim

**Affiliations:** ^1^Department of Medical Biochemistry, Oslo University Hospital, Oslo, Norway; ^2^Unger-Vetlesen Institute, Lovisenberg Diakonale Hospital, Oslo, Norway; ^3^Department of Plastic and Reconstructive Surgery, Oslo University Hospital, Norway; ^4^Institute of Oral Biology, Faculty of Dentistry, University of Oslo, Oslo, Norway; ^5^Institute for Surgical Research, Oslo University Hospital, Oslo, Norway; ^6^Faculty of Medicine, University of Oslo, Oslo, Norway; ^7^Department of Ophthalmology, Oslo University Hospital, Oslo, Norway; ^8^Department of Clinical Medicine, Faculty of Medicine, University of Bergen, Bergen, Norway; ^9^Department of Ophthalmology, Stavanger University Hospital, Stavanger, Norway; ^10^Department of Ophthalmology, Drammen Hospital, Drammen, Norway; ^11^Department of Ophthalmology, Sørlandet Sykehus, Arendal, Arendal, Norway

## Abstract

There is a need to optimize storage conditions to preserve cell characteristics during transport of cultured cell sheets from specialized culture units to distant hospitals. In this study, we aimed to explore a method to identify additives that diminish the decrease in the viability of stored undifferentiated epidermal cells using multifactorial design and an automated screening procedure. The cultured cells were stored for 7–11 days at 12°C in media supplemented with various additives. Effects were evaluated by calcein staining of live cells as well as morphology. Twenty-six additives were tested using (1) a two-level factorial design in which 10 additives were added or omitted in 64 different combinations and (2) a mixture design with 5 additives at 5 different concentrations in a total of 64 different mixtures. Automated microscopy and cell counting with Fiji enabled efficient processing of data. Significant regression models were identified by Design-Expert software. A calculated maximum increase of live cells to 37 ± 6% was achieved upon storage of cell sheets for 11 days in the presence of 6% glycerol. The beneficial effect of glycerol was shown for epidermal cell sheets from three different donors in two different storage media and with two different factorial designs. We have thus developed a high throughput screening system enabling robust assessment of live cells and identified glycerol as a beneficial additive that has a positive effect on epidermal cell sheet upon storage at 12°C. We believe this method could be of use in other cell culture optimization strategies where a large number of conditions are compared for their effect on cell viability or other quantifiable dependent variables.

## 1. Introduction

The first example of regenerative medicine occurred in 1984 with life-saving transplantation of cultured epidermal cells sheets (CES) to two boys with severe large area burns [1]. CES continues to be commonly used in clinics to regenerate skin in cases of large area burns and chronic hard-to-heal ulcers [2]. They also have potential for use as an autologous epithelial cell layer, which can be substituted to regenerate other types of epithelia in the body. Examples include use of CES in regeneration of damaged cornea [3] and urethra [4] in animal models. It is expected that transplantation of a high percentage of viable cells in the CES is important to ensure successful integration following transplantation.

The demand for CES may increase as the field of regenerative medicine becomes more established. However, the need for increased safety regulations means that culture facilities are becoming progressively centralized to meet strict requirements [5]. This opens up a gap between patients at regional centers and specialized culture facilities. In the present study, we aimed to develop a system for storage and transport that would make high quality CES available for an extended period of time. This would allow expanded delivery to clinics regionally and internationally [6]. In addition, short-term storage accommodates patient needs in cases of critical life-threatening large area burns. It allows a larger window for use of large batches of autologous CES where successive surgeries are necessary. It also gives flexibility in the timing of surgery depending on the status of the patient. In addition, the flexibility provided by optimal storage conditions could lead to more efficient use of hospital resources during preparation of CES and when scheduling surgery time. A stable storage period provides the further benefit of a window for extensive quality control testing [7].

Earlier studies have shown that storage of CES at 12°C is optimal for preserving undifferentiated epidermal cell characteristics and provides close to 100% viability over a one-week storage period [8, 9]. However, viability falls to ~60% over a two-week storage period [9, 10]. Identification of one or more additives that significantly improve cell viability and morphology could extend storage time beyond one week and improve the quality of CES delivered for surgery. To test a large number of additives, we chose a factorial design approach over one-factor-at-a-time testing making use of a software package, Design-Expert® developed by Stat-Ease Inc. This reduced the number of experiments necessary to test all possible interactions between the selected test additives. The design of experiment approach also allowed automation of data analysis to a large degree, thus further improving accuracy and efficiency.

## 2. Materials and Methods

### 2.1. Overview of Strategy

Additives were selected for testing based on a literature search for substances shown to combat oxidative damage and/or increase cell proliferation. Relevant additives were first tested as a two-level multiple combination, in which sets of 10 different additives (Tables 1 and 2) were evaluated in experiments 1 and 2 for combinatorial effects, using epidermal cells from two different donors, respectively. Cultured confluent CES were stored for 7 days at 12°C in experiments 1 and 2. The following experiments, 3 and 4 used a mixture design, testing storage for 11 days using 5 additives at 5 levels with epidermal cells from two different donors, respectively. Glycerol, the best candidate from experiment 1, was carried forward for further verification in all experiments.

### 2.2. Supplies

Medium for epidermal cells (CnT-Prime) was purchased from CELLnTEC (Bern, Switzerland). Trypsin-EDTA, glycerol, 4-(2-hydroxyethyl)-1-piperazineethanesulfonic acid (HEPES), and NaHCO_3_ were purchased from Sigma-Aldrich (St. Louis, MO). Nunclon Δ surface multidishes, pipettes, and other routine plastics were obtained from VWR International (West Chester, PA). Phosphate-buffered saline (PBS) and Minimum Essential Medium (MEM) were from Life Technologies (Carlsbad, CA). Icilin and menthol were from Santa Cruz Biotechnology (Heidelberg, Germany). Suppliers for tested additives are presented in Table 1. Storage Mat III for 96 Well Plates (Corning, NY) were used for air-tight covering of microtiter plates at storage. Live and dead cells were assessed by the LIVE/DEAD® Viability/Cytotoxity Kit (molecular probes, OR).

### 2.3. Cell Culture

Human tissue was used in accordance with the Declaration of Helsinki and was harvested from adult female donors undergoing breast reduction or abdominal reduction surgery (abdominoplasty) following written informed consent. Local ethical committee approval was obtained for the use of tissue from the Department of Plastic Surgery, Oslo University Hospital, Oslo, Norway [2013/815/REK]. Cells were obtained as described [8], seeded (6000 cells/well) in serum-free CnT-Prime medium on Collagen IV coated 96-well Nunclon Δ surface multidishes, and cultured in a 5% CO_2_ incubator at 37°C for 5–7 days to obtain a confluent monolayer. Culture medium was changed every two days.

### 2.4. Use of Factorial Design to Evaluate Optimal Concentrations and Combinations of Additives

Various additives were evaluated by one or both of the design types (2^10^ or 5^5^) as specified in Table 2 and explained below. Design type 2^10^ was used in experiments 1A, 1B, and 2. It comprised a setup with 10 different additives, each at two levels for evaluation of combinatorial effects. Design-Expert® software enabled a 64-run (64 individual mixtures), fractional factorial single-plate design. Thus, all combinations of 10 additives at a time could be evaluated while reducing the number of a full factorial experiment (2^10^ = 1024 possible combinations) to 64. This experiment setup is shown in Table S1. Design type 5^5^ was used in experiments 3 and 4. It was comprised of a mixture factorial design, in which 5 additives at 5 different combinations were evaluated by response surface methodology [11] and a central composite design [12]. While a full factorial experiment would need 5^5^ = 3125 experimental groups, we could design a single plate setup with 64 runs (mixtures) with the aid of the Design-Expert® software. This design setup is presented in Table S2.

### 2.5. Cell Storage

Cells were stored with various combinations of additives for seven or eleven days before assessment of the additives' effect on viability and morphology. A Biomek 4000 automated workstation (robot) was used to mix concentrated solutions of additives or MEM storage medium (MEM with 25 mM HEPES, 300 mg/l NaHCO_3_, and 50 *μ*g/ml gentamycin) into 64 wells of a 96-well reservoir plate. From the reservoir plate, the storage media containing various additives were distributed into 64 wells of 96-well plates (*n* = 6) that contained confluent epidermal cells to be stored. Each well was made air tight by covering with Storage Mat III™ and the plates were stored at 12°C for 7 or 11 days. The standard deviation of the temperature in each storage container was ±0.4°C as demonstrated previously [13]. Following storage, the medium was replaced with CnT-Prime medium, and cells were allowed to equilibrate at 37°C and 5% CO_2_ for 3 h before further analysis in order to include assessment of any potential damage incurred upon rewarming [9].

### 2.6. Quantitative Analysis of Live and Dead Cells

After equilibration of stored cells for 3 hours at 37°C in growth medium, live and dead cells were stained with calcein or ethidium, respectively, using a LIVE/DEAD® kit according to the manufacturer's instructions. After staining, photomicrographs of the cultures were captured using a Nikon Eclipse Ti fluorescence microscope with a DS-Qi1 black-and-white camera and a motorized stage. Photographs were taken automatically using the “capture multipoint” function and autofocusing with “steps in range.” The exposure length and gain was maintained at a constant level for all samples, and the fluorescence intensities of the fluorochromes were within the dynamic range of the camera. The number of live or dead cells per square unit (picture) was automatically quantified using Fiji (National Institutes of Health, Bethesda, MD, USA) [14]. See supplementary for script and details. The numbers were further analyzed by the Design-Expert® software (Stat-Ease Inc., Minneapolis, MN) as described below.

Measurements of dead cells gave conflicting results, which is likely due to the fact that only attached dead cells can be counted. Thus, a significant part of dead cells could not be counted since the cell layer had disrupted and cells had detached from the plate.

### 2.7. Assessment of Morphology

The morphology of stored cells was evaluated based on pictures from each run. The cells were given marks between 1 and 4 based on subjective evaluation of cell integrity, confluence, density, and shape and were evaluated blindly by two persons. Average marks from up to 6 parallel plates were subjected to analysis by Design-Expert® software as described below.

### 2.8. Statistical Methods

Various regression models (linear, quadratic, reduced quadratic, special cubic, and cubic) were tested with the aid of Design-Expert® software to best describe the results, and recommended models were selected based on highest significance. In experiment 1, a sum of squares model was selected as the best fit for viability data, while a quadratic model was best fit to data on morphology. For the remaining experiments, a reduced quadratic model showed the best fit. The theory behind mixture design and analysis is described by Cornell [15]. In short, the measured response (e.g., number of live cells after storage) depends on the proportion of each ingredient (additive) in the mixture and not the amount of mixture. The blending of additives, each at multiple proportions of the mixture in different runs/mixtures, allows prediction of favorable/unfavorable individual additives as well as calculation of synergistic or antagonistic effects between different additives. Significant effects of the various additives were tested within the Design-Expert® software using analysis of variance (ANOVA). Pearson correlation was used to test relative effects of additives in two different storage media, and Student's *t*-test was used to evaluate the difference in number of live cells after storage in the two media.

## 3. Results

### 3.1. Experiment 1

In experiment 1, effects of 10 additives were compared in two different basic storage media, CnT Prime (experiment 1A) and Minimum Essential Medium (MEM) (experiment 1B), previously shown to be beneficial for storage experiments [16, 17]. Relative effect of additives were similar across the 64 tested combinations between the two storage media (*r* = 0.72), but storage in MEM resulted in 30.5% more live cells after storage (*p* value: 1.0 × 10^−12^) as compared to CnT Prime. Thus, MEM was used as the storage medium in all following experiments.

#### 3.1.1. Live Cells

**Figure 1:**
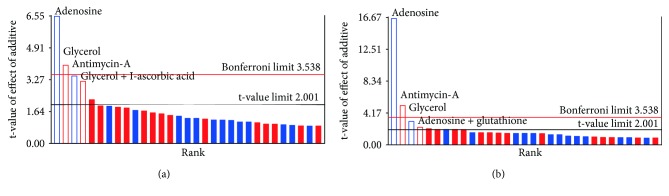
Charts displaying the relative effects of the various additives on number of live cells (a) or morphology (b). The *y*-axis shows *t* values of the absolute effects on the cells upon addition of various additives (*x*-axis). Blue or red bars: additives calculated to have negative or positive effects, respectively, on the number of live cells compared to storage without any additive. Filled bars = significance > 0.05 (not significant) No-fill bars = significance < 0.05 (significant). The figures are reconstructed in Prism Graph Pad from output in Design-Expert to improve clarity.

Using the sum of squares model, cell viability results (Table 3) showed that glycerol, adenosine, and antimycin-A alone, as well as the combination of glycerol and l-ascorbic acid, had a significant effect on the number of viable cells.  Figure 1(a) shows the relative magnitude and direction of effect. Adenosine and antimycin-A had a significant negative effect on cell survival (*p* < 0.0001 and *p* = 0.0010, respectively). Glycerol had a significant positive effect on cell survival that was maintained after Bonferroni correction for multiple testing (*p* = 0.0002) [18]. Glycerol combined with L-ascorbic acid showed significant positive effects (*p* = 0.0022) but did not sustain Bonferroni correction for multiple testing. Simulation by the Point Prediction tool in Design-Expert (Table S3) indicated that 1% glycerol in MEM would increase the number of live cells by 1.8% compared to MEM basic storage medium without additives. The predicted increase in live cells with the combination of glycerol and L-ascorbic acid was 8.0%. It should be noted that none of the combinations tested in Experiment 1 included just MEM + glycerol or MEM + glycerol + L-ascorbic acid; thus, these are calculated effects.

#### 3.1.2. Morphology

The quadratic model predicted that glycerol was the single additive that resulted in better morphology compared to MEM basic storage medium alone (*p* = 0.0049, data not shown). However, the effect did not sustain adjustment for multiple testing by the Bonferroni method (Figure 1(b)). As found for analysis of live cells, adenosine and antimycin A showed markedly negative effects on cell morphology.

### 3.2. Experiment 2

For experiment 2, eight more additives were tested in addition to glycerol at 0.75% and 3% using epidermal cells from a different donor (Tables 1 and 2). No significant effects on live or dead cells or morphology were detected.

### 3.3. Experiment 3

For experiment 3, a mixture design with 5 additives at 5 different concentrations was used. This experiment was designed to identify the optimal concentration of glycerol in combination with 4 other candidate additives during storage for 11 days (Tables 1 and 2). Glycerol was included in some of the mixtures at varying concentrations from 2.0 to 6.0%.

#### 3.3.1. Live Cells

The reduced quadratic mixture model predicted that glycerol, fenoldopam mesylate, carnosine, and DMSO added as single additives significantly affected the number of live cells (Table 4). Using the Point Prediction tool, the maximum concentration of glycerol (6%) was calculated to result in a 35% increase in viable cells (Table 5). Fenoldopam mesylate as a single additive gave a predicted result of a 5% increase in the number of viable cells at its optimal concentration (11 *μ*M). Glycerol in combination with fenoldopam mesylate was also significantly positively associated with an increased number of live cells after 11 days of storage. The maximum number of live cells was predicted at 6% glycerol +8 *μ*M fenoldopam mesylate, with an increase in cell viability of 37% (Table 5). Carnosine worked against the positive effect of glycerol, while DMSO had a positive and negative effect (<2%) at low and high concentrations, respectively.

#### 3.3.2. Morphology

The reduced quadratic mixture model predicted that carnosine and fenoldopam mesylate, separately and together, had a beneficial effect on morphology (Table 6). In line with the effect on the number of live cells, glycerol in combination with fenoldopam mesylate had a positive effect on morphology, while glycerol in combination with carnosine showed a negative effect. LIF and DMSO abolished the positive effect of fenoldopam mesylate, while carnosine showed a positive effect on morphology with LIF but negative with DMSO.

### 3.4. Experiment 4

For experiment 4, we used the same experimental design as in experiment 3 (a mixture design with 5 additives at 5 different concentrations) with cells from the same donor as used in experiment 2 but at a later passage (P3). Cell sheets were stored for 11 days. Glycerol was included in some of the mixtures at varying concentrations from 3.3 to 10.0%.

#### 3.4.1. Viability

A reduced quadratic mixture model was the best fit for the cell viability data (*p* < 0.0001), and analysis of the 5 substances as single additives indicated that only glycerol had a positive effect (*p* = 0.0004, Table 7). Glycerol in combination with any of the other 4 additives had a significant negative effect. Simulation by the Point Prediction tool in experiment 4 indicated that the theoretical optimal concentration of glycerol as a single additive was 3.6%, at which the number of live cells was predicted to increase by 7.1% (Table 8).

#### 3.4.2. Morphology

**Figure 2:**
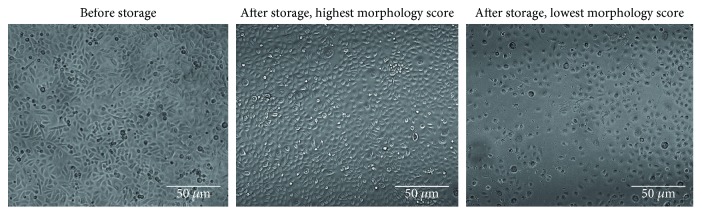
Typical light microscopy images illustrating morphology of cells before and after storage for 11 days. The cells were primary epidermal cells stored at passage 4.

The only single additives predicted to result in a significant positive effect on morphology in experiment 4 were carnosine and fenoldopam mesylate. Glycerol as a single additive did not reach significance but had a positive effect in combination with fenoldopam mesylate. Combinations of carnosine with fenoldopam mesylate or LIF were also calculated to have a positive effect on morphology. Carnosine + glycerol had a negative effect on morphology, while the combination of carnosine with DMSO had a positive effect on morphology. Using the Point Prediction tool for morphology data, the maximal predicted number of live cells was achieved at 18.4 mM carnosine + 15 uM fenoldopam mesylate + 6% glycerol + 40 ng/ml LIF, increasing the morphology ranking by 52%.  Figure 2 shows an example of cells prior to storage as well as high and low morphology ranked wells after 11 days of storage. Cells with high morphology rating look very similar to nonstored cells.

## 4. Discussion

We have successfully developed a high throughput method for screening the individual and combined effect of media additives on CES during storage. We tested various combinations and concentrations of additives for their effect on survival and morphology of undifferentiated epidermal cells during storage at 12°C. In this study, we used two different multifactorial designs based on Design-Expert® software.

Accurate, high throughput analysis was enabled by automated photography of calcein-stained cells followed by automated cell counting using the Multiple Image Processor function within Fiji [14]. Visual evaluation of morphology was also performed. A limited number of studies have used multifactorial design to screen for various cell traits; examples include Jakobsen et al., who analyzed combinations of 5 different factors in a full 2^5^ factorial design for optimization of chondrogenesis of mesenchymal stem cells with levels of mRNAs encoding chondrogenic markers as output [19]. Another example was optimizing Drosophila organ culture conditions employing a mixture design on undifferentiated cells as organ proxy [20]. However, to our knowledge, this is the first study combining Fiji-facilitated automatic counting of cells with factorial design to achieve a much higher throughput. In general, visual inspection of morphology confirmed cell viability measurements, while measurement of dead cells gave conflicting results, possibly due to detachment/lysis of dead cells, thereby making them undetectable.

Regression analysis of results from two different factorial design experimental setups predicted that glycerol increases survival of stored CES. Results were consistent across three out of four experiments using these experimental setups using two different storage media formulations. The additives fenoldopam mesylate and possibly L-ascorbic acid enhanced the effect of glycerol. Though the predicted optimal concentration of glycerol varied between experiments, possibly due to the use of cells from different donors, the models indicated a consistent positive effect. We attribute the lack of effect of glycerol in experiment 2 to the short storage time (7 days) and the use of cells from an early passage (p2). Cells may not have been stressed enough to get observable results. This conclusion is supported by experiment 4, in which glycerol clearly had a beneficial effect on viability of cells from a later passage from the same donor during a longer 11-day storage period.

Glycerol has been shown to enter cells via aquaglyceroporins, a subclass of aquaporins including AQP3 which is abundant and important in epidermal cells [21, 22]. Although glycerol has been widely used as a cryoprotectant, its use in preserving the essential qualities of cultured cell sheets at above freezing temperatures pretransplantation has not been explored. It has been reported that pig skin allografts can be successfully preserved with 85% glycerol when kept at 4°C [23, 24]. Studies have shown that a lower concentration of glycerol (<6% in cell culture or intratesticular injection of a 10% glycerol solution) suppresses proliferation [25, 26]. Wiebe and Dinsdale conducted experiments using several cell lines and showed that glycerol completely suppressed proliferation when used at a concentration of 4%–8% (depending on cell type) [27]. Furthermore, replacement of glycerol medium with glycerol-free medium resulted in full recovery of proliferation rate following exposure to 4% glycerol but only partial recovery (65%) following exposure to 10–12% glycerol. Thus, glycerol may shut down important energy demanding cellular processes, thereby also reducing oxidative damage and promoting cell survival.

Fenoldopam mesylate is a dopamine 1 receptor (D1R) agonist shown to stimulate robust activation of AMP-activated protein kinase (AMPK) in various cell types [28, 29]. Silencing of AMPK results in reduced mitochondrial and eNOS (endothelial NO synthase) content, reduced cell proliferation, increased accumulation of ROS, and apoptosis [30]. As shown for fenoldopam mesylate, L-ascorbic acid has a well-documented effect on reduction of oxidative damage to cells [31, 32]. Furthermore, L-ascorbic acid has been shown to have a positive effect on proliferation of porcine corneal endothelial cells [33]. We therefore cannot exclude that the beneficial effect of L-ascorbic acid on increasing the number of viable cells was due to proliferation during storage.

The optimal predicted concentration of glycerol and the other recommended additives indicated by Design-Expert© remain to be tested. Future planned studies include testing these recommendations with a large number of donors and using a larger CES area. This experimental setup was considered too large for inclusion in the present initial work. The results indicate a positive effect of glycerol on cell viability in cells from three different donors and two different basic storage media. Consistent results showing a positive effect of glycerol across three experiments suggest that using a multifactorial design may be an innovative and reliable method to discover the effect of new formulations in similar setups with various goals and using different cell types. Furthermore, use of a robot for mixing storage media and automated cell counting ensured less variation due to human handling.

## 5. Conclusions

We have developed a high throughput screening system enabling robust assessment of live cells and identified glycerol as a beneficial additive that has a positive effect on cell survival in CES during storage at 12°C. We believe this method could be of use in other cell culture optimization strategies where a large number of groups are compared for their effect on cell viability or other quantifiable factors.

## Figures and Tables

**Table 1:** Tested additives and suppliers.

Additive	Concentration	Supplier
Acetovanillone	50 *μ*M	Sigma-Aldrich (St. Louis, MO)
Adenosine	5 mM	Sigma-Aldrich (St. Louis, MO)
Allopurinol	1 mM	Sigma-Aldrich (St. Louis, MO)
Antimycin A	20 nM	Sigma-Aldrich (St. Louis, MO)
Aspirin (acetylsalicylic acid)	133–400 *μ*g/ml	Sigma-Aldrich (St. Louis, MO)
ATP (adenosine triphosphate)	67–200 *μ*M	Sigma-Aldrich (St. Louis, MO)
Dimethyl (S)-(−)-malate	2,5 mM	Sigma-Aldrich (St. Louis, MO)
DMSO (dimethyl sulfoxide)	0.07–0.20%	Sigma-Aldrich (St. Louis, MO)
Fenoldopam mesylate	6.7–20 *μ*M	Sigma-Aldrich (St. Louis, MO)
L-Gluthatione	3 mM	Sigma-Aldrich (St. Louis, MO)
Glycerol	7.5–100 mg/ml	Sigma-Aldrich (St. Louis, MO)
Hydrocortizone	3 ng/ml	Sigma-Aldrich (St. Louis, MO)
Icilin	0.33–1 *μ*M	Santa Cruz Biotechnology (Heidelberg, Germany)
Insulin	5 *μ*g/ml	Sigma-Aldrich (St. Louis, MO)
Lactic acid	13–40 mM	Sigma-Aldrich (St. Louis, MO)
L-Ascorbic acid	50 *μ*g/ml	Sigma-Aldrich (St. Louis, MO)
L-carnosine	13.3–40 mM	Sigma-Aldrich (St. Louis, MO)
Leukemia inhibitory factor (LIF)	13–40 ng/ml	Sigma-Aldrich (St. Louis, MO)
LiCl	1 mM	Merck (Oslo, Norway)
Melatonin	0.67–2 nM	Calbiochem (San Diego, CA)
Menthol	16.7–50 *μ*M	Santa Cruz Biotechnology (Heidelberg, Germany).
Methyl pyruvate	1 mM	Sigma-Aldrich (St. Louis, MO)
N-(2-Mercaptopropionyl)glycine (NMPG)	100 *μ*M	Sigma-Aldrich (St. Louis, MO)
N-Acetyl-L-cysteine	1 mM	Sigma-Aldrich (St. Louis, MO)
Sodium pyruvate	10 mM	Sigma-Aldrich (St. Louis, MO)
Taurine	20 mM	Sigma-Aldrich (St. Louis, MO)

**Table 2:** Summary of experiments.

Exp. #	Design type	Donor/source	Storage time	Tested additives
1A Storage in MEM	2^10^	♀age 53, abdomen	7 days	Glycerol (1%)
				L-Ascorbic acid
				Allopurinol
				Sodium pyruvate
				Adenosine
				Taurine
1B Storage in CnT Prime				L-Glutathione
				Hydrocortizone
				LiCl
				Antimycin-A
2	2^10^	♀age 40, abdomen	7 days	Glycerol 0.75%
				Glycerol 3%
				Icilin
				Menthol
				Dimethyl (S)-(−)-malate
				Methyl pyruvate
				N-Acetyl-L-cysteine
				Insulin
				Acetovanillone
				N-(2-Mercaptopropionyl)glycine (NMPG)
3	5^5^	♀ age 47, breast	11 days	L-carnosine
				DMSO
				Fenoldopam mesylate
				Glycerol (up to 6%)
				LIF
4	5^5^	♀age 40, abdomen	11days	Glycerol (up to 10%)
				Aspirin
				Melatonin
				Lactic acid
				ATP

**Table 3:** Effect of different additives on the number of live cells after one-week storage.

Additives	*F* value	*p* value prob > *F*	Effect
Adenosine (5 mM)	42.31	<0.0001	−
L-Ascorbic acid (50 *μ*g/ml)	0.20	0.6575	
Glycerol (1%)	16.19	0.0002	+
Antimycin A (20 nM)	12.09	0.0010	−
L-Ascorbic acid and Glycerol (50 ug/ml and 1%, respectively)	10.25	0.0022	+

*F* value: test for comparing model variance with residual variance; *p* value prob > *F*: probability of observed *F* value if the null hypothesis is true (small value call for rejection of the null hypothesis). Only additives involved in a significant or positive effect on viability are shown. Parentheses indicate tested concentrations of the respective additives. All additives were added to MEM basic storage medium.

**Table 4:** Reduced quadratic mixture model for the number of live cells.

Additives	*F* value	*p* value prob > *F*	Effect
Carnosine (40 mM)	5.66	0.0213	+
DMSO (0.2%)	10.88	0.0018	−
Fenoldopam mesylate (10 *μ*M)	6.79	0.0121	+
Glycerol (6%)	5.11	0.0283	+
Carnosine + DMSO (0.2%)	15.78	0.0002	−
Carnosine + glycerol	27.15	<0.0001	−
Carnosine + LIF (40 ng/ml)	3.27	0.0768	
DMSO + glycerol	4.55	0.0380	−/+
Fenoldopam mesylate + LIF	3.11	0.0839	

*F* value: test for comparing model variance with residual variance; *p* value prob > *F*: probability of observed *F* value if the null hypothesis is true (small value call for rejection of the null hypothesis). Parentheses indicate maximal tested concentrations of the relevant additives and not necessarily the optimal levels.

**Table 5:** Estimated number of live cells by the Design-Expert Point Prediction tool.

Mean	Std dev	95% CI low	95% CI high
Predicted number of live cells with no additives			
1814	149	1665	1963
Predicted number of live cells at 6.0% glycerol			
2420	149	2232	2608
Predicted number of live cells at 6% glycerol + 8uM fenoldopam mesylate			
2461	149	2312	2609

**Table 6:** Morphology evaluations using the reduced quadratic mixture model.

Additives	*F* value	*p* value prob > *F*	Effect
Carnosine (40 mM)	9.31	0.0037	+
Fenoldopam mesylate (10 *μ*M)	48.90	<0.0001	+
Carnosine + DMSO (0.2%)	8.74	0.0048	−
Carnosine + fenoldopam mesylate	30.96	<0.0001	+
Carnosine + glycerol (6%)	9.20	0.0039	−
Carnosine + LIF (40 ng/ml)	8.19	0.0062	+
DMSO + fenoldopam mesylate	69.17	<0.0001	−
Fenoldopam mesylate + glycerol	64.85	<0.0001	+
Fenoldopam mesylate + LIF	25.24	<0.0001	−

*F* value: test for comparing model variance with residual variance; *p* value prob > *F*: probability of observed *F* value if the null hypothesis is true (small value call for rejection of the null hypothesis) Parentheses indicate maximal tested concentrations of the relevant additives and not necessarily the optimal levels.

**Table 7:** Reduced quadratic mixture model for the number of live cells.

Additives	*F* value	*p* value prob > *F*	Effect
Glycerol (10%)	14.41	0.0004	+
Lactic acid	2.87	0.0964	−
Glycerol + aspirin (400 *μ*g/ml)	5.89	0.0187	−
Glycerol + ATP (200 *μ*M)	12.34	0.0009	−
Glycerol + lactic acid (40 mM)	15.78	0.0002	−
Glycerol + melatonin (2 nM)	8.72	0.0047	−

*F* value: test for comparing model variance with residual variance; *p* value prob > *F*: probability of observed *F* value if the null hypothesis is true (small value call for rejection of the null hypothesis) Parentheses indicate maximal tested concentrations of the relevant additives and not necessarily the optimal levels.

**Table 8:** Predicted number of live cells using the Design-Expert Point Prediction tool.

Mean	Std dev	95% CI low	95% CI high
Predicted number of live cells with no additives			
924	139	796	1053
Predicted number of live cells at 3.8% glycerol			
990	139	875	1105

## Data Availability

The data used to support the findings of this study are available from the corresponding author upon request.

## Supplementary Materials

Supplementary Materials Table S1: setup for 10 additives at two-level multiple combination testing. Table S2: setup for mixture design. Table S3: number of live cells with glycerol additive as predicted by Design-Expert.
