# Supplementary material for: High Throughput Screening of Additives Using Factorial Design to Promote Survival of Stored Cultured Epithelial Sheets
Source: Stem Cells Int. 2018 Nov 18;2018:6545876. doi: 10.1155/2018/6545876 (PMC6276401; doi:10.1155/2018/6545876)
Supplement: Supplementary Materials — Table S1: setup for 10 additives at two-level multiple combination testing. Table S2: setup for mixture design. Table S3: number of live cells with glycerol additive as predicted by Design-Expert. [file 6545876.f1.docx]

**Supplementary Materials**

**High Throughput Screening of Additives Using Factorial Design to Promote Survival of Stored Cultured Epithelial Sheets**

Sjur Reppe^1,2^, Catherine Joan Jackson^1,3,4^, Håkon Ringstad^1^, Kim Alexander Tønseth^3,5,6^, Hege Bakke^1^, Jon Roger Eidet^7^, Tor Paaske Utheim^1,3,6,7,8,9,10,11^

^1^Department of Medical Biochemistry, Oslo University Hospital, Oslo, Norway

^2^Unger-Vetlesen Institute, Lovisenberg Diaconal Hospital, Oslo, Norway

^3^Department of Plastic and Reconstructive Surgery, Oslo University Hospital, Norway

^4^Institute of Oral Biology, Faculty of Dentistry, University of Oslo, Oslo, Norway

^5^Institute for Surgical Research, Oslo University Hospital, Oslo, Norway

^6^Faculty of Medicine, University of Oslo, Oslo, Norway

^7^Department of Ophthalmology, Oslo University Hospital, Oslo, Norway

^8^Department of Clinical Medicine, Faculty of Medicine, University of Bergen, Bergen, Norway

^9^Department of Ophthalmology, Stavanger University Hospital, Stavanger, Norway

^10^Department of Ophthalmology, Drammen Hospital, Drammen, Norway

^11^Department of Ophthalmology, Sørlandet Sykehus, Arendal, Arendal, Norway

| **Table S1.** Set up for 10 additives at 2 levels multiple combination testing | | | | | | | | | | | | | | | | | | |  |
| --- | --- | --- | --- | --- | --- | --- | --- | --- | --- | --- | --- | --- | --- | --- | --- | --- | --- | --- | --- |
| **Run** | **allopurinol** | | **pyruvate** | | **adenosine** | **taurine** | | **gluthatione** | | **L-ascorbic acid** | | **glycerol** | | **hydro-cortisone** | **LiCl** | | **antimycin A** | | |
| **1** | 1 | | 1 | | -1 | 1 | | -1 | | -1 | | -1 | | 1 | 1 | | 1 | | |
| **2** | 1 | | 1 | | -1 | -1 | | -1 | | 1 | | 1 | | 1 | 1 | | 1 | | |
| **3** | 1 | | 1 | | 1 | -1 | | -1 | | -1 | | -1 | | -1 | -1 | | -1 | | |
| **4** | 1 | | -1 | | 1 | 1 | | -1 | | 1 | | -1 | | 1 | -1 | | 1 | | |
| **5** | -1 | | 1 | | 1 | 1 | | -1 | | 1 | | -1 | | 1 | 1 | | -1 | | |
| **6** | 1 | | 1 | | -1 | 1 | | -1 | | 1 | | -1 | | 1 | -1 | | -1 | | |
| **7** | 1 | | 1 | | 1 | 1 | | -1 | | 1 | | 1 | | -1 | -1 | | -1 | | |
| **8** | -1 | | 1 | | 1 | 1 | | -1 | | -1 | | -1 | | 1 | -1 | | 1 | | |
| **9** | 1 | | 1 | | -1 | 1 | | 1 | | -1 | | -1 | | -1 | -1 | | -1 | | |
| **10** | 1 | | 1 | | 1 | 1 | | 1 | | 1 | | 1 | | 1 | 1 | | 1 | | |
| **11** | -1 | | 1 | | 1 | -1 | | 1 | | -1 | | 1 | | -1 | -1 | | 1 | | |
| **12** | 1 | | 1 | | 1 | -1 | | -1 | | 1 | | -1 | | -1 | 1 | | 1 | | |
| **13** | -1 | | 1 | | -1 | -1 | | -1 | | -1 | | -1 | | -1 | 1 | | -1 | | |
| **14** | -1 | | 1 | | 1 | 1 | | 1 | | 1 | | -1 | | -1 | -1 | | 1 | | |
| **15** | -1 | | 1 | | 1 | -1 | | -1 | | -1 | | 1 | | 1 | 1 | | -1 | | |
| **16** | -1 | | -1 | | -1 | -1 | | 1 | | 1 | | 1 | | -1 | 1 | | 1 | | |
| **17** | 1 | | -1 | | -1 | -1 | | -1 | | 1 | | -1 | | -1 | 1 | | -1 | | |
| **18** | -1 | | 1 | | 1 | 1 | | 1 | | -1 | | -1 | | -1 | 1 | | -1 | | |
| **19** | 1 | | -1 | | 1 | -1 | | 1 | | 1 | | 1 | | -1 | -1 | | 1 | | |
| **20** | -1 | | -1 | | -1 | 1 | | -1 | | -1 | | -1 | | 1 | -1 | | -1 | | |
| **21** | -1 | | -1 | | -1 | 1 | | 1 | | 1 | | -1 | | -1 | -1 | | -1 | | |
| **22** | -1 | | -1 | | -1 | 1 | | 1 | | -1 | | -1 | | -1 | 1 | | 1 | | |
| **23** | 1 | | 1 | | -1 | -1 | | 1 | | 1 | | 1 | | -1 | -1 | | -1 | | |
| **24** | 1 | | -1 | | 1 | 1 | | 1 | | 1 | | -1 | | -1 | 1 | | -1 | | |
| **25** | -1 | | -1 | | -1 | -1 | | 1 | | -1 | | 1 | | -1 | -1 | | -1 | | |
| **26** | -1 | | -1 | | 1 | -1 | | 1 | | -1 | | -1 | | 1 | -1 | | -1 | | |
| **27** | -1 | | 1 | | -1 | 1 | | -1 | | -1 | | 1 | | -1 | -1 | | 1 | | |
| **28** | 1 | | -1 | | 1 | 1 | | 1 | | -1 | | -1 | | -1 | -1 | | 1 | | |
| **29** | -1 | | 1 | | -1 | 1 | | 1 | | 1 | | 1 | | 1 | -1 | | 1 | | |
| **30** | 1 | | 1 | | -1 | -1 | | 1 | | -1 | | 1 | | -1 | 1 | | 1 | | |
| **31** | -1 | | -1 | | 1 | -1 | | -1 | | -1 | | -1 | | -1 | 1 | | 1 | | |
| **32** | 1 | | -1 | | -1 | -1 | | 1 | | 1 | | -1 | | 1 | -1 | | 1 | | |
| **33** | -1 | | 1 | | -1 | -1 | | 1 | | 1 | | -1 | | 1 | 1 | | -1 | | |
| **34** | 1 | | -1 | | 1 | 1 | | -1 | | -1 | | -1 | | 1 | 1 | | -1 | | |
| **35** | 1 | | -1 | | 1 | -1 | | -1 | | 1 | | 1 | | 1 | 1 | | -1 | | |
| **36** | 1 | | -1 | | 1 | -1 | | 1 | | -1 | | 1 | | -1 | 1 | | -1 | | |
| **37** | -1 | | -1 | | 1 | 1 | | -1 | | 1 | | 1 | | -1 | 1 | | 1 | | |
| **38** | 1 | | -1 | | -1 | 1 | | 1 | | -1 | | 1 | | 1 | -1 | | 1 | | |
| **39** | 1 | | -1 | | -1 | 1 | | -1 | | -1 | | 1 | | -1 | 1 | | -1 | | |
| **40** | -1 | | 1 | | 1 | -1 | | -1 | | 1 | | 1 | | 1 | -1 | | 1 | | |
| **41** | -1 | | -1 | | 1 | -1 | | -1 | | 1 | | -1 | | -1 | -1 | | -1 | | |
| **42** | -1 | | 1 | | -1 | -1 | | 1 | | -1 | | -1 | | 1 | -1 | | 1 | | |
| **43** | -1 | | 1 | | -1 | 1 | | -1 | | 1 | | 1 | | -1 | 1 | | -1 | | |
| **44** | 1 | | -1 | | -1 | -1 | | 1 | | -1 | | -1 | | 1 | 1 | | -1 | | |
| **45** | -1 | | -1 | | 1 | 1 | | 1 | | 1 | | 1 | | 1 | -1 | | -1 | | |
| **46** | 1 | | -1 | | -1 | -1 | | -1 | | -1 | | -1 | | -1 | -1 | | 1 | | |
| **47** | -1 | | -1 | | -1 | -1 | | -1 | | -1 | | 1 | | 1 | 1 | | 1 | | |
| **48** | 1 | | 1 | | -1 | -1 | | -1 | | -1 | | 1 | | 1 | -1 | | -1 | | |
| **49** | -1 | | -1 | | 1 | 1 | | -1 | | -1 | | 1 | | -1 | -1 | | -1 | | |
| **50** | 1 | | 1 | | 1 | 1 | | -1 | | -1 | | 1 | | -1 | 1 | | 1 | | |
| **51** | -1 | | -1 | | -1 | -1 | | -1 | | 1 | | 1 | | 1 | -1 | | -1 | | |
| **52** | -1 | | -1 | | 1 | -1 | | 1 | | 1 | | -1 | | 1 | 1 | | 1 | | |
| **53** | -1 | | 1 | | -1 | 1 | | 1 | | -1 | | 1 | | 1 | 1 | | -1 | | |
| **54** | 1 | | 1 | | -1 | 1 | | 1 | | 1 | | -1 | | -1 | 1 | | 1 | | |
| **55** | -1 | | 1 | | 1 | -1 | | 1 | | 1 | | 1 | | -1 | 1 | | -1 | | |
| **56** | 1 | | -1 | | -1 | 1 | | -1 | | 1 | | 1 | | -1 | -1 | | 1 | | |
| **57** | 1 | | 1 | | 1 | 1 | | 1 | | -1 | | 1 | | 1 | -1 | | -1 | | |
| **58** | 1 | | -1 | | -1 | 1 | | 1 | | 1 | | 1 | | 1 | 1 | | -1 | | |
| **59** | 1 | | 1 | | 1 | -1 | | 1 | | 1 | | -1 | | 1 | -1 | | -1 | | |
| **60** | 1 | | 1 | | 1 | -1 | | 1 | | -1 | | -1 | | 1 | 1 | | 1 | | |
| **61** | -1 | | -1 | | 1 | 1 | | 1 | | -1 | | 1 | | 1 | 1 | | 1 | | |
| **62** | -1 | | 1 | | -1 | -1 | | -1 | | 1 | | -1 | | -1 | -1 | | 1 | | |
| **63** | -1 | | -1 | | -1 | 1 | | -1 | | 1 | | -1 | | 1 | 1 | | 1 | | |
| **64** | 1 | | -1 | | 1 | -1 | | -1 | | -1 | | 1 | | 1 | -1 | | 1 | | |
| The table shows runs (well number in 96 well plate) to be supplemented with respective additives (1) and runs in which the respective additives are omitted (-1). | | | | | | | | | | | | | | | | | | |  |
| **Table S2.** Set up for mixture design | | | | | | | | | | | | | | | |  |  |  |  |
| **Run** | | **MEM storage medium** | | | **Comp 1** | | **Comp 2** | | **Comp 3** | | **Comp 4** | | | **Comp 5** | |  |  |  |  |
| **1** | | 50 | | | 10 | | 10 | | 10 | | 10 | | | 10 | |  |  |  |  |
| **2** | | 66.67 | | | 0 | | 0 | | 13.33 | | 20 | | | 0 | |  |  |  |  |
| **3** | | 53.33 | | | 6.67 | | 20 | | 0 | | 20 | | | 0 | |  |  |  |  |
| **4** | | 70 | | | 10 | | 0 | | 20 | | 0 | | | 0 | |  |  |  |  |
| **5** | | 66.67 | | | 0 | | 20 | | 0 | | 13.33 | | | 0 | |  |  |  |  |
| **6** | | 80 | | | 20 | | 0 | | 0 | | 0 | | | 0 | |  |  |  |  |
| **7** | | 50 | | | 10 | | 10 | | 10 | | 10 | | | 10 | |  |  |  |  |
| **8** | | 60 | | | 6.67 | | 13.33 | | 20 | | 0 | | | 0 | |  |  |  |  |
| **9** | | 46.67 | | | 6.67 | | 20 | | 0 | | 6.67 | | | 20 | |  |  |  |  |
| **10** | | 73.33 | | | 0 | | 0 | | 20 | | 6.67 | | | 0 | |  |  |  |  |
| **11** | | 33.33 | | | 20 | | 20 | | 20 | | 6.67 | | | 0 | |  |  |  |  |
| **12** | | 50 | | | 10 | | 10 | | 10 | | 10 | | | 10 | |  |  |  |  |
| **13** | | 86.67 | | | 0 | | 6.67 | | 6.67 | | 0 | | | 0 | |  |  |  |  |
| **14** | | 60 | | | 0 | | 6.67 | | 0 | | 13.33 | | | 20 | |  |  |  |  |
| **15** | | 33.33 | | | 20 | | 20 | | 6.67 | | 0 | | | 20 | |  |  |  |  |
| **16** | | 20 | | | 20 | | 20 | | 20 | | 0 | | | 20 | |  |  |  |  |
| **17** | | 60 | | | 6.67 | | 0 | | 13.33 | | 0 | | | 20 | |  |  |  |  |
| **18** | | 20 | | | 6.67 | | 20 | | 20 | | 20 | | | 13.33 | |  |  |  |  |
| **19** | | 100 | | | 0 | | 0 | | 0 | | 0 | | | 0 | |  |  |  |  |
| **20** | | 30 | | | 20 | | 10 | | 20 | | 20 | | | 0 | |  |  |  |  |
| **21** | | 40 | | | 20 | | 0 | | 0 | | 20 | | | 20 | |  |  |  |  |
| **22** | | 70 | | | 0 | | 20 | | 0 | | 0 | | | 10 | |  |  |  |  |
| **23** | | 60 | | | 10 | | 0 | | 10 | | 10 | | | 10 | |  |  |  |  |
| **24** | | 60 | | | 10 | | 0 | | 10 | | 10 | | | 10 | |  |  |  |  |
| **25** | | 53.33 | | | 20 | | 13.33 | | 0 | | 13.33 | | | 0 | |  |  |  |  |
| **26** | | 30 | | | 20 | | 0 | | 20 | | 10 | | | 20 | |  |  |  |  |
| **27** | | 33.33 | | | 6.67 | | 0 | | 20 | | 20 | | | 20 | |  |  |  |  |
| **28** | | 66.67 | | | 20 | | 0 | | 0 | | 0 | | | 13.33 | |  |  |  |  |
| **29** | | 40 | | | 13.33 | | 6.67 | | 20 | | 0 | | | 20 | |  |  |  |  |
| **30** | | 60 | | | 6.67 | | 0 | | 0 | | 20 | | | 13.33 | |  |  |  |  |
| **31** | | 46.67 | | | 6.67 | | 20 | | 0 | | 6.67 | | | 20 | |  |  |  |  |
| **32** | | 53.33 | | | 0 | | 20 | | 13.33 | | 13.33 | | | 0 | |  |  |  |  |
| **33** | | 50 | | | 10 | | 10 | | 10 | | 10 | | | 10 | |  |  |  |  |
| **34** | | 10 | | | 20 | | 20 | | 10 | | 20 | | | 20 | |  |  |  |  |
| **35** | | 33.33 | | | 0 | | 20 | | 20 | | 6.67 | | | 20 | |  |  |  |  |
| **36** | | 53.33 | | | 20 | | 20 | | 0 | | 0 | | | 6.67 | |  |  |  |  |
| **37** | | 50 | | | 10 | | 10 | | 10 | | 10 | | | 10 | |  |  |  |  |
| **38** | | 53.33 | | | 0 | | 13.33 | | 20 | | 0 | | | 13.33 | |  |  |  |  |
| **39** | | 46.67 | | | 0 | | 20 | | 0 | | 20 | | | 13.33 | |  |  |  |  |
| **40** | | 46.67 | | | 0 | | 0 | | 13.33 | | 20 | | | 20 | |  |  |  |  |
| **41** | | 50 | | | 10 | | 10 | | 10 | | 10 | | | 10 | |  |  |  |  |
| **42** | | 46.67 | | | 20 | | 0 | | 13.33 | | 20 | | | 0 | |  |  |  |  |
| **43** | | 100 | | | 0 | | 0 | | 0 | | 0 | | | 0 | |  |  |  |  |
| **44** | | 30 | | | 0 | | 20 | | 10 | | 20 | | | 20 | |  |  |  |  |
| **45** | | 50 | | | 10 | | 10 | | 10 | | 10 | | | 10 | |  |  |  |  |
| **46** | | 73.33 | | | 0 | | 0 | | 20 | | 6.67 | | | 0 | |  |  |  |  |
| **47** | | 80 | | | 13.33 | | 0 | | 0 | | 6.67 | | | 0 | |  |  |  |  |
| **48** | | 40 | | | 6.67 | | 13.33 | | 20 | | 20 | | | 0 | |  |  |  |  |
| **49** | | 70 | | | 0 | | 20 | | 0 | | 0 | | | 10 | |  |  |  |  |
| **50** | | 50 | | | 10 | | 10 | | 10 | | 10 | | | 10 | |  |  |  |  |
| **51** | | 10 | | | 20 | | 10 | | 20 | | 20 | | | 20 | |  |  |  |  |
| **52** | | 80 | | | 0 | | 0 | | 0 | | 0 | | | 20 | |  |  |  |  |
| **53** | | 66.67 | | | 13.33 | | 20 | | 0 | | 0 | | | 0 | |  |  |  |  |
| **54** | | 30 | | | 20 | | 20 | | 0 | | 20 | | | 10 | |  |  |  |  |
| **55** | | 20 | | | 20 | | 20 | | 20 | | 13.33 | | | 6.67 | |  |  |  |  |
| **56** | | 86.67 | | | 0 | | 0 | | 6.67 | | 0 | | | 6.67 | |  |  |  |  |
| **57** | | 80 | | | 0 | | 13.33 | | 0 | | 6.67 | | | 0 | |  |  |  |  |
| **58** | | 53.33 | | | 20 | | 0 | | 20 | | 0 | | | 6.67 | |  |  |  |  |
| **59** | | 80 | | | 0 | | 0 | | 0 | | 20 | | | 0 | |  |  |  |  |
| **60** | | 86.67 | | | 0 | | 6.67 | | 6.67 | | 0 | | | 0 | |  |  |  |  |
| **61** | | 53.33 | | | 0 | | 0 | | 20 | | 20 | | | 6.67 | |  |  |  |  |
| **62** | | 46.67 | | | 20 | | 20 | | 13.33 | | 0 | | | 0 | |  |  |  |  |
| **63** | | 50 | | | 20 | | 10 | | 0 | | 0 | | | 20 | |  |  |  |  |
| **64** | | 60 | | | 0 | | 6.67 | | 0 | | 13.33 | | | 20 | |  |  |  |  |
| The table shows volume (%) to be added of MEM storage medium or MEM with additives at 5 times maximal concentration (Comp 1-5). | | | | | | | | | | | | | | | |  |  |  |  |

| **Table S3.** Number of live cells with glycerol additive as predicted by Design Expert | |
| --- | --- |
| **Predicted number of live cells with no additives** | |
| Mean | Std Dev |
| 4858 | 435 |
| **Predicted number of live cells at 1% glycerol** | |
| 4948 | 435 |
| **Predicted number of live cells at 1% glycerol + 50 ug/ml L-ascorbic acid** | |
| 5248 | 435 |
| The Point Prediction Tool in Design Expert was used for predictions | |

**Automatic cell counting**

The following script was used for automatic quantification of the number of live cells from tiff formatted pictures and run under the command “Multiple Image Processor” (subheading under main command “Process”:

| Script | Comments |
| --- | --- |
| run("Invert");  run("8-bit");  //run("Brightness/Contrast...");  setMinAndMax(150, 250);  setAutoThreshold("Default");  //run("Threshold...");  //setThreshold(0, 171);  setOption("BlackBackground", false);  run("Convert to Mask");  run("Watershed");  run("Watershed");  run("Analyze Particles...", "size=5-Infinity circularity=0.70-1.00 show=[Bare Outlines] display clear include summarize"); | Inverts the image (white to black)  Transform the image type from 16 bit to 8 bit  Opens the Brightness/Contrast command  Sets the level of minimal and maximal display range to 150 and 250, respectively,  Sets Auto Threshold to “default”  Activates Threshold function and selects particles of default threshold  Sets the white to black threshold limits to 0 and 171, respectively (the range from white to black is 255)  Prevents the background from being black  Converts the image to black and white based on the current threshold settings  Increases space between close particles (cells)  Further Increases space between close particles (cells)  Counts particles restricted to those of size ≥5 and circularity 0.7-1.00 and outlines the counted particles |
| For quantification of dead cells “size=5” may be changed to “size=2” (at the end of the script) to reflect the smaller size of the stained nucleus. The numbers were further analyzed by the Design-Expert® software (Stat-Ease, Inc., Minneapolis, MN) as described. | |
